# Estimating the direct effect of human papillomavirus vaccination on the lifetime risk of screen‐detected cervical precancer

**DOI:** 10.1002/ijc.33207

**Published:** 2020-07-28

**Authors:** Federica Inturrisi, Birgit I. Lissenberg‐Witte, Nienke J. Veldhuijzen, Johannes A. Bogaards, Guglielmo Ronco, Chris J. L. M. Meijer, Johannes Berkhof

**Affiliations:** ^1^ Amsterdam UMC, Vrije Universiteit Amsterdam, Epidemiology and Data Science, Amsterdam Public Health Amsterdam The Netherlands; ^2^ The Leprosy Research Initiative Amsterdam The Netherlands; ^3^ Centre for Infectious Disease Control National Institute for Public Health and the Environment Bilthoven The Netherlands; ^4^ International Agency for Research on Cancer Lyon France; ^5^ Amsterdam UMC, Vrije Universiteit Amsterdam, Pathology, Cancer Center Amsterdam Amsterdam The Netherlands

**Keywords:** cervical intraepithelial neoplasia (CIN), HPV‐based screening, human papillomavirus (HPV), lifetime risk, prophylactic vaccination

## Abstract

Birth cohorts vaccinated against human papillomavirus (HPV) are now entering cervical cancer screening. Assessment of (pre)cancer (CIN3+) risk is needed to assess the residual screening need in vaccinated women. We estimated the lifetime (screen‐detected) CIN3+ risk under five‐yearly primary HPV screening between age 30 and 60, using HPV genotyping and histology data of 21,287 women participating in a screening trial with two HPV‐based screening rounds, 5 years apart. The maximum follow‐up after an HPV‐positive test was 9 years. We re‐estimated the CIN3+ risk after projecting direct vaccine efficacy for the bivalent and the nonavalent HPV vaccines, assuming life‐long protection. The lifetime CIN3+ risk was 4.1% (95% confidence interval 3.5‐4.9) and declined by 53.5% and 70.5% after bivalent vaccination without and with cross‐protection, respectively, translating into a residual lifetime CIN3+ risk of 1.9% (1.4‐2.4) and 1.2% (0.9‐1.5). The CIN3+ risk declined by 88.5% after nonavalent vaccination, translating into a residual lifetime CIN3+ risk of 0.5% (0.2‐0.7). The latter risk increased to 1.6% when vaccine protection only lasted until the first screening round at age 30. Among HPV‐positive women with abnormal adjunct cytology, the nine‐year CIN3+ risk was 16.9% (8.7‐32.4) after nonavalent vaccination. In conclusion, HPV vaccination will lead to a strong decline in the lifetime CIN3+ risk and the remaining absolute CIN3+ risk will be very low. Primary HPV testing combined with adjunct cytology at five‐year intervals still seems feasible even after nonavalent vaccination, although unlikely to be cost‐effective. Our results support a de‐intensification of screening programs in settings with high vaccination coverage.

## Abstract 2

**What's new?**

Human papillomavirus (HPV)‐based screening and vaccination are key strategies for early detection and prevention of cervical intraepithelial neoplasia grade 3 and cancer (CIN3+). Here the authors estimated the lifetime screen‐detected CIN3+ risk expected under different HPV vaccination scenarios using a novel statistical, data‐driven approach that deals with multiple‐type HPV infections. The model estimated that the lifetime CIN3+ risks under five‐yearly primary HPV screening will become very low in vaccinated women, in particular when protection is life‐long and provided beyond genotypes 16 and 18. These CIN3+ risks are important when defining new extended screening intervals for vaccinated cohorts.

**Abbreviations**
2/4/9vHPV: bivalent/quadrivalent/nonavalent HPV vaccine
CI: confidence interval
CIN (2/3+): cervical intraepithelial neoplasia (grade 2/3 or worse)
HPV: human papillomavirus
PALGA: nationwide network and registry of histo‐ and cytopathology in the Netherlands, Dutch for “Pathologisch‐Anatomisch Landelijk Geautomatiseerd Archief”
POBASCAM: Population‐based Screening Study Amsterdam

## 1 INTRODUCTION

In the past decade, cervical cancer prevention has changed drastically. Prophylactic human papillomavirus (HPV) vaccination has been implemented in immunization programs in over 70 countries by now.^1^ The first‐generation HPV vaccines, that is, the bivalent and quadrivalent vaccines, have shown to provide nearly 100% protection against HPV16‐ and HPV18‐positive cervical intraepithelial neoplasia grade 2 (CIN2) or 3 (CIN3) and to provide partial cross‐protection against several other high‐risk HPV types.^2^, ^3^ The more recent nonavalent HPV vaccine has been shown to be non‐inferior to the first generation vaccines with respect to protection against HPV16 and HPV18, and also provides nearly 100% protection against five other high‐risk HPV types (HPV types 31, 33, 45, 52 and 58).^4^, ^5^ In several countries, HPV testing is being considered or has been implemented in the screening program either in combination with cytology testing or as a single, primary test.^6^ HPV‐based screening has shown to lead to earlier detection of CIN3 and cancer (CIN3+) and to provide greater protection against invasive cervical cancer than cytology‐based screening.^7^ Countries that replaced cytology‐based screening by HPV‐based screening also reduced the screening intensity to limit the number of colposcopy referrals and CIN2 treatments and further revisions are anticipated for vaccinated cohorts. An imminent question is therefore how to integrate the new primary and secondary prevention options into one comprehensive program.

Vaccination and screening programs target different age groups. Routine vaccination is delivered to girls aged 9 to 14 years whereas organized screening is usually initiated between age 20 and 30. Hence, the first cohorts that were offered vaccination are now entering screening age or have completed one round of screening. In the Netherlands, the first vaccinated birth cohort, eligible for catch‐up vaccination in 2009, will enter the screening program in 2023. The screening need for vaccinated women will crucially depend on their residual risk of cervical precancer. The first results (from Australia, Denmark, Sweden, the United Kingdom and the United States) show a substantial reduction in CIN2/3 in vaccinated women as compared to unvaccinated women,^8^, ^9^, ^10^ supporting a revision of the screening guidelines for vaccinated cohorts.^11^, ^12^, ^13^ Screening strategies for vaccinated women can be evaluated in a formal way by cost‐effectiveness analyses. Most cost‐effectiveness analyses indicated that a combination of vaccination and HPV‐based screening with intervals of at least 5 years, from age 25 or 30 to age 60 or 65, is cost‐effective in women vaccinated with a bivalent/quadrivalent vaccine.^11^, ^12^, ^14^, ^15^, ^16^ An important limitation of cost‐effectiveness analyses is that they rely on complex, mathematical models for describing progression of an HPV infection to CIN3+. A complementary, data‐driven approach for evaluating screening strategies is to estimate CIN3+ risk directly from longitudinal data,^17^, ^18^ where a low CIN3+ risk after a negative screen supports an extension of the screening interval.^19^

The aim of our study is to estimate the lifetime risk of (screen‐detected) CIN3+ and of CIN2 and worse (CIN2+), using the Population‐based Screening Study Amsterdam (POBASCAM) in which 44,102 Dutch women receiving their regular screening invitation were randomized to one or two rounds of HPV‐based screening with an interval of 5 years.^20^ HPV genotyping was applied to all HPV‐positive samples. The POBASCAM study is particularly suitable for studying the impact of vaccination because the Dutch program uses screening intervals of 5 years and starts at age 30, which is a non‐intensive screening strategy that is currently being considered in several countries for HPV‐vaccinated cohorts. As herd effects will be limited for the first vaccinated cohorts,^21^, ^22^ we here focus on the direct benefit of HPV vaccination for vaccinated women in terms of residual screening need.

## 2 MATERIALS AND METHODS

### 2.1 Study population

**FIGURE 1:**
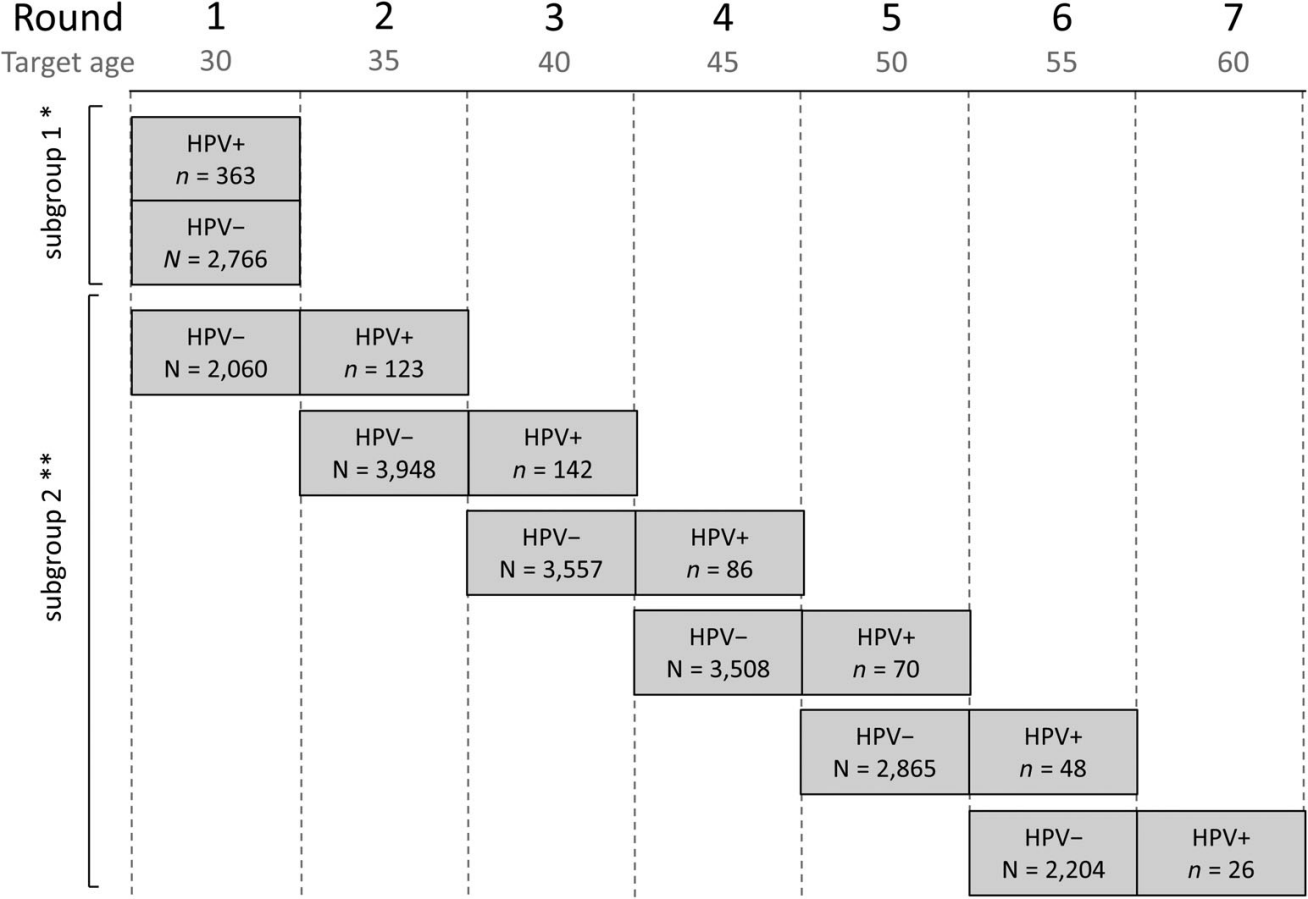
Study data. *Intervention group only. **Intervention and control group, only women who attended the second screening round of the POBASCAM trial

POBASCAM is a population‐based randomized screening trial conducted in setting of the cervical cancer screening program in the Netherlands with the enrolment from January 1999 until September 2002 and the design has been published previously.^20^, ^23^ Briefly, 44,102 women attending routine cervical cancer screening visits (age 29‐61) were invited to participate. Eligible, consenting women were randomly assigned (1:1) to the intervention (HPV and cytology co‐testing) or control group (cytology‐only). HPV results in the control arm were blinded and not used for clinical management. During the second round 5 years later, all women were managed using co‐testing. For our study, we included women from two partially overlapping subgroups: women aged 29 to 33 years from the intervention group (subgroup 1, N = 3,129) and women aged 29 to 58 years from both the intervention and control group having an HPV‐negative test result at baseline, no CIN2+ in the baseline round and a co‐test result at the next screening round after 5 years (subgroup 2, N = 18,637) (Figure 1). In line with the POBASCAM trial analysis,^20^ screening results within 4 years after baseline were classified as results from the baseline round whereas results between 4 and 9 years after baseline were classified as results from the next round.

The study is registered at the International Trial Register (ISRCTN20781131) and is now finished.

### 2.2 Procedures

Physician‐collected endocervical brush material for HPV testing was stored in collection medium (5 mL phosphate‐buffered saline and 0.5% thiomersal) and tested by the Department of Pathology at the VU University Medical Center. Duplicate GP5+/6+ PCR‐enzyme immunoassay followed by reverse line blot analysis on positive samples was carried out as described previously.^24^ A mixture of PCR‐probes was used for the detection of 14 high‐risk HPV types (HPV types 16, 18, 31, 33, 35, 39, 45, 51, 52, 56, 58, 59, 66 and 68). Endocervical brush material was also read for cytology and grouped according to the CISOE‐A classification^25^ which can be converted to the 2001 Bethesda system.^26^

Histological follow‐up was obtained from all four participating laboratories, and data were also tracked through the nationwide pathology database (PALGA Foundation, Houten, the Netherlands).^27^ We collected 9 years of follow‐up after an HPV‐positive test result. Histology was examined locally and classified (in order of increasing severity) as no lesion, CIN grade 1, 2, 3 or invasive cancer according to international criteria.^28^ Adenocarcinoma in situ was included in the CIN3 group. CIN2 and CIN3 histology was sufficient to treat women by a loop electrosurgical excision procedure.

### 2.3 Statistical methods

In the Netherlands, women are invited for screening in the calendar year at which they turn 30, 35, 40, 45, 50, 55 and 60 years of age (Figure 1). Actual age at screening may be 1 year younger than the target age, and we categorized age in the following groups: 29 to 33, 34 to 38, 39 to 43, 44 to 48, 49 to 53, 54 to 58, and 59 to 63 years.

We estimated the reduction in cumulative risk of CIN3+ and CIN2+ up to (target) age 60 (lifetime screen‐detected CIN3+ and CIN2+ risks), under different vaccine scenarios. We also calculated the CIN3+ and CIN2+ risk after an HPV‐positive result or after an HPV‐positive, cytology abnormal result.

The estimation method uses the following natural history assumptions: (I) the risk of a new type‐specific HPV infection only depends on age and is not influenced by cohort effects or previous HPV infections, (II) CIN2/3+ are caused by a high‐risk HPV infection, (III) a woman can have multiple type‐specific CIN2/3+ at the same time, and (IV) the type‐specific CIN2/3+ risks in an HPV‐positive woman are not influenced by coinfections with other HPV genotypes^29^, ^30^ and are constant beyond age 30.

Following Assumption I, we estimated the probabilities of a new HPV‐positive result in Rounds 2 to 7 using data collected over two screening rounds (HPV‐negative baseline screen and one screen at the next round). We denote the probability of having a new HPV‐positive test result at the *i*th screening round (*i* = 2, …, 7) by *P*_*i*_ and denote the probability of a prevalent HPV infection at Round 1 by *P*_1_.

We estimated CIN3+ risks among HPV‐positive women in two steps. First, we estimated the HPV type‐specific CIN3+ risks among HPV type‐positive women by maximizing the likelihood under Assumptions III and IV.^29^ We used 9 years of follow‐up after an HPV‐positive test for the calculation of CIN3+ risks. Women without CIN3+ detected during follow‐up were assumed not to have progressed to CIN3+. Second, we pooled the type‐specific CIN3+ risks on the basis of the observed genotype distribution among HPV‐positive women. Following Assumption IV, we estimated separate CIN3+ risks for prevalent infections in Round 1 (target age 30) and incident infections in Rounds 2 to 7 (target age > 30). These are denoted by respectively *C*_1_ and *C*_2_ and formulas can be found in the Supporting Information.

The cumulative risk of CIN3+ up to age 60 (i.e. lifetime risk) is then:

$$\text{Lifetime}\;\mathrm{CIN}3+\;\text{risk}=P_{1}\times C_{1}+\sum_{i=2}^{7}\prod_{j=1}^{i-1}\left(1-P_{j}\right)\times P_{i}\times C_{2}.$$

We also estimated CIN3+ risk after abnormal adjunct cytology in HPV‐positive women. For this purpose, we estimated the type‐specific risks of abnormal adjunct cytology and of combined abnormal adjunct cytology and CIN3+.^29^ As before, we pooled the type‐specific risks according to the observed genotype distribution in HPV‐positive women to get overall risks. The overall risks of abnormal adjunct cytology and of combined abnormal adjunct cytology and CIN3+ are denoted by *Q*_1_ and *D*_*1*_ for Round 1 and by *Q*_*2*_ and *D*_*2*_ for Rounds 2 to 7. The CIN3+ risks after abnormal adjunct cytology are then equal to:

$$\mathrm{CIN}3+\;\text{risk}\;\text{after}\;\text{abnormal}\;\text{adjunct}\;\text{cytology}\;q=D_{q}/Q_{q},\;q=1,2.$$

For further details, see the Supporting Information.

#### 2.3.1 Effect of vaccination

We assumed direct vaccine‐induced protection against CIN3+ and CIN2+ to be mediated by a reduction in the type‐specific infection probabilities, with life‐long efficacy against HPV vaccine types. We assumed the bivalent/quadrivalent vaccines (2/4vHPV) to have 100% efficacy against HPV16 and HPV18 based on reported efficacies against CIN3+ and CIN2+ of 98% to 100%.^2^, ^31^, ^32^ We also assumed the bivalent vaccine to provide some cross‐protection against other high‐risk HPV types (2vHPV + cross‐protection). The cross‐protection efficacies were obtained by pooling results of two clinical trials.^33^, ^34^ Only efficacies that were significant at the 0.1 level after pooling were included, yielding efficacies of 74.2% for HPV31, 42.8% for HPV33, 73.4% for HPV45 and 10.4% for HPV52. Similar to the vaccine types, we assumed cross‐protection to be life‐long based on 8 years of follow‐up after vaccination.^35^, ^36^ Finally, we assumed the nonavalent vaccine (9vHPV) to provide, in addition to the 100% efficacy against HPV16 and HPV18, 96.3% efficacy against high‐risk HPV types 31, 33, 45, 52 and 58.^37^

To assess the impact of life‐long vaccine efficacy on the lifetime CIN3+ risk, we performed a sensitivity analysis in which we assumed vaccination to confer protection only up to screening Round 1 at age 30, both for HPV vaccine types and cross‐protective types.

To assess uncertainty around reported efficacies against non16/18 HPV types, additional sensitivity analyses were performed. For the bivalent vaccine, we replaced the base‐case pooled efficacies by single study efficacy estimates. More specifically, we used the following significant (*P* = 0.05, one‐sided) cross‐protective efficacies reported by single studies: Wheeler et al^33^ (76.5% HPV31, 44.6% HPV33, 73.5% HPV45, 16.4% HPV51); Herrero et al^34^ (64.7% HPV31, 73.0% HPV45); Kavanagh et al^38^ (93.8% HPV31, 79.1% HPV33, 82.6% HPV45); Bogaards et al^35^ (66.0% HPV31, 41.0% HPV33, 40.0% HPV35, 81.0% HPV45, 36.0% HPV52, 30.0% HPV58) and Lehtinen et al^39^ (75.5% HPV31, 43.3% HPV33, 80.6% HPV45). For the nonavalent vaccine, we replaced the reported efficacies against non16/18 types by the lower bound of the 95% confidence interval (CI): 79.5%.^37^

For each of the three vaccine scenarios (2/4vHPV, 2vHPV + cross‐protection and 9vHPV), we re‐estimated *P*_1_, …, *P*_7_ and *C*_1_, *C*_2_, *D*_1_, *D*_2_, *Q*_1_ and *Q*_2_ (see Supporting Information) and substituted the new estimates in the formulas for the lifetime CIN3+ and CIN2+ risks.

Maximum likelihood estimates are reported with 95% CI, calculated from 1,000 nonparametric bootstrap samples of the POBASCAM study data. Analyses were performed in R, version 3.4.2 (R foundation for Statistical Computing, Vienna, Austria).

In the following, we present results for CIN3+. Results for CIN2+ can be found in the Supporting Information. Results of the intermediate estimates needed for the estimation of the lifetime risks of CIN3+ and CIN2+ can be found in Figures S1 and S2.

## 3 RESULTS

### 3.1 Study cohort characteristics

Three‐hundred and seventy three (11.6%) out of 3,129 women with target age 30 (aged 29 to 33 years) from the intervention group were HPV‐positive in Round 1 (prevalent infections, subgroup 1) (Figure 1). Four‐hundred and ninety five (2.7%) out of 18,637 women with target ages 30 to 55 from both the intervention and control study group were HPV‐positive in the following round after a negative test result (incident infections, subgroup 2). During the entire follow‐up period of maximum 9 years after a positive HPV result, 87 CIN3+ and 126 CIN2+ were diagnosed among women with a prevalent infection at target age 30 and 44 CIN3+ and 89 CIN2+ were diagnosed among women with an incident HPV infection at older age.

### 3.2 Effect of vaccination on the lifetime CIN3+ risk

**FIGURE 2:**
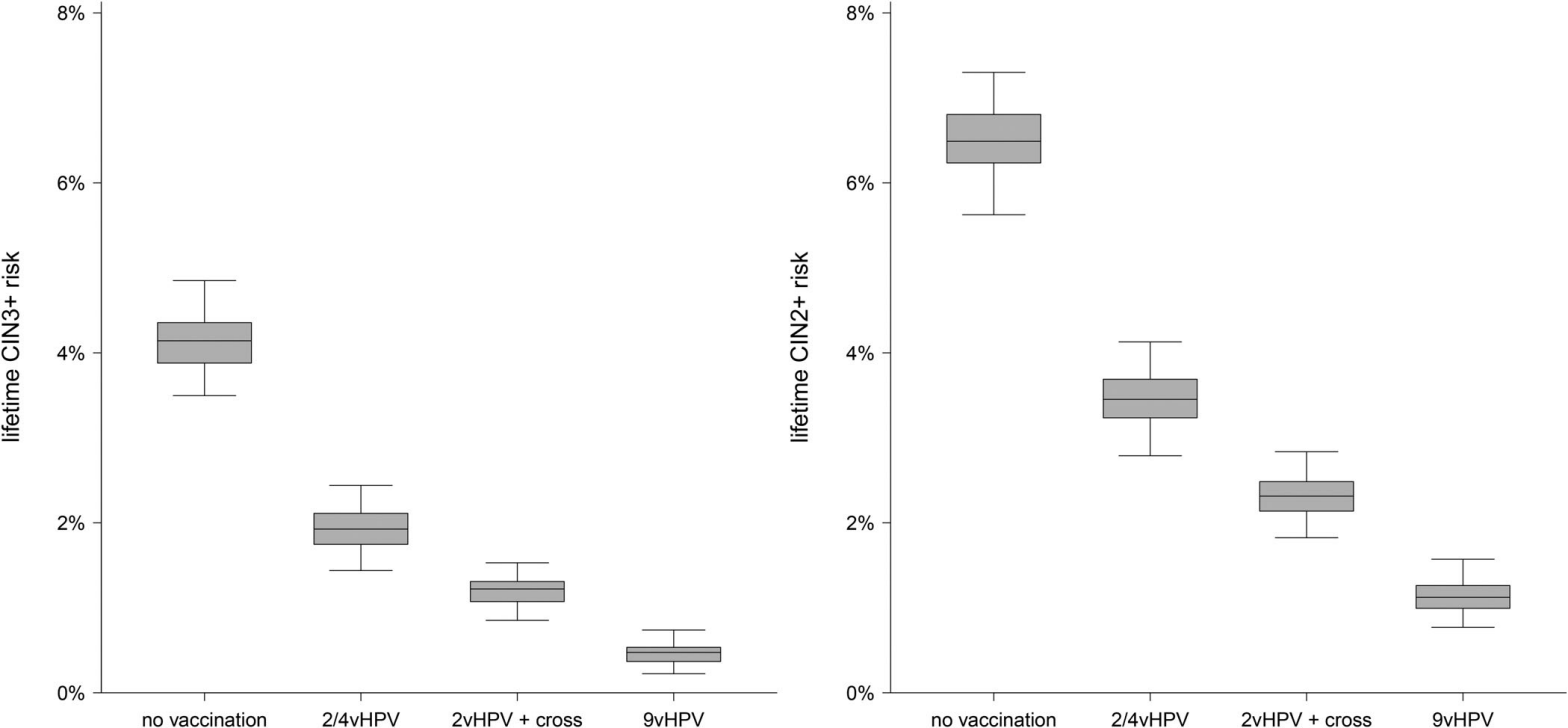
Effect of vaccination on the lifetime risks of CIN3+ (left) and CIN2+ (right)

Under no vaccination, the lifetime CIN3+ risk was 4.1% (95% CI = 3.5‐4.9) (Figure 2, Table S1). The lifetime risk decreased to 1.9% (1.4‐2.4) under bivalent/quadrivalent vaccination without cross‐protection and to 1.2% (0.9‐1.5) under bivalent vaccination with cross‐protection. Finally, under nonavalent vaccination, the lifetime CIN3+ risk was only 0.5% (0.2‐0.7). In relative terms, bivalent/quadrivalent vaccination without cross‐protection, bivalent vaccination with cross‐protection and nonavalent vaccination yielded declines of 53.5% (43.7‐62.2), 70.5% (64.4‐78.0) and 88.5% (82.4‐94.3) as compared to no vaccination. The incremental CIN3+ risk reduction was 0.7% (0.5‐1.0) for the addition of direct cross‐protective effects to the bivalent vaccine and 0.7% (0.5‐1.0) for the comparison of nonavalent vaccination to bivalent vaccination with cross‐protection. This indicates that direct vaccine effect against non16/18 HPV‐related CIN3+ is about half as large for the bivalent vaccine as compared to the nonavalent vaccine.

The lifetime CIN3+ risks reduced by more than 50% after an HPV‐negative test in Round 1 (Table S1). Among those women, the residual lifetime CIN3+ risk was 1.4% (1.0‐1.9) without vaccination, 0.8% (0.5‐1.2) under bivalent/quadrivalent vaccination without cross‐protection, 0.5% (0.3‐0.7) under bivalent vaccination with cross‐protection, and 0.2% (0.1‐0.4) under nonavalent vaccination.

When vaccine efficacy was set to confer protection only up to Round 1 at age 30, the lifetime CIN3+ risk was 2.5% (1.9‐3.1) under bivalent/quadrivalent vaccination without cross‐protection, 2.1% (1.6‐2.6) under bivalent vaccination with cross‐protection and 1.6% (1.2‐2.1) under nonavalent vaccination (Table S1). In relative terms, bivalent/quadrivalent vaccination without cross‐protection, bivalent vaccination with cross‐protection and nonavalent vaccination yielded declines of 40.1% (31.4‐49.2), 49.7% (41.5‐58.5), and 60.6% (51.7‐69.0) as compared to no vaccination.

The sensitivity analyses for bivalent vaccination with cross‐protective effects as reported in single studies yielded lifetime CIN3+ risks between 0.9% (Kavanagh study) and 1.5% (Herrero study). The lifetime CIN3+ risks for nonavalent vaccination with efficacy against non16/18 types set at the lower 95% CI bound was 0.7%.

### 3.3 Effect of vaccination on the CIN3+ risk in HPV‐positive women

**FIGURE 3:**
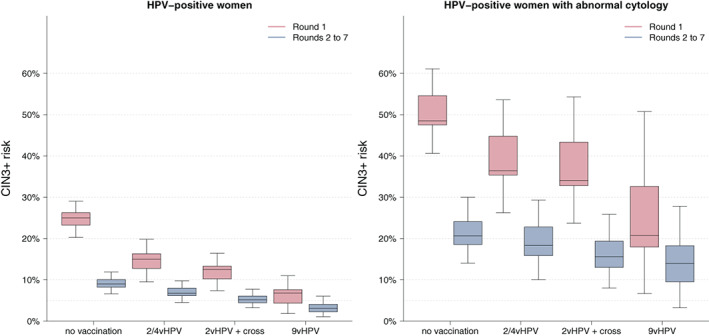
Effect of vaccination on the CIN3+ risks in HPV‐positive women (left) and in HPV‐positive women with abnormal adjunct cytology (right)

Under no vaccination, the CIN3+ risk was 25.0% (20.3‐29.1) in women with a prevalent HPV infection in Round 1 and 9.0% (6.6‐11.9) in women with an incident HPV infection in Rounds 2 to 7 (Figure 3, Table S2). The CIN3+ risk in Round 1 and in Rounds 2 to 7 decreased, respectively, to 15.0% (9.5‐19.8) and 6.8% (4.5‐9.8) under bivalent/quadrivalent vaccination without cross‐protection, to 12.5% (7.4‐16.4) and 5.1% (3.2‐7.7) under bivalent vaccination with cross‐protection, and to 6.8% (1.8‐11.0) and 3.1% (1.1‐6.1) under nonavalent vaccination.

In HPV‐positive women, CIN3+ risks increased substantially when adjunct cytology was abnormal (Figure 3, Table S3). Under no vaccination, the CIN3+ risk was 52.0% (40.6‐61.1) in Round 1 and 20.3% (14.1‐30.0) in Rounds 2 to 7. Vaccination had a limited effect on the CIN3+ risk which, under nonavalent vaccination, was still 28.2% (6.6‐50.8) in Round 1 and 13.9% (3.2‐27.8) in Rounds 2 to 7.

### 3.4 End‐point CIN2+

For end‐point CIN2+, lifetime risk was 6.6% (5.6‐7.3) in unvaccinated women and decreased to 1.1% (0.8‐1.6) under nonavalent vaccination (Figure 2). The relative declines under the different vaccination scenarios were slightly smaller as compared to those for end‐point CIN3+, both under life‐long protection and protection up to Round 1 at age 30 (Table S1). Under no vaccination, the CIN2+ risks in HPV‐positive women were 34.6% (29.9‐39.7) in Round 1 and 17.9% (14.6‐21.3) in Rounds 2 to 7 (Table S2). The CIN2+ risks in HPV‐positive women with abnormal cytology were 65.9% (54.5‐73.2) in Round 1 and 40.2% (32.0‐50.9) in Rounds 2 to 7 (Table S3). The relative declines in CIN2+ risks in HPV‐positive women and in HPV‐positive women with abnormal cytology were slightly smaller than those for CIN3+ in Round 1 and considerably smaller than those for CIN3+ in Rounds 2 to 7. Under nonavalent vaccination, the CIN2+ risk in HPV‐positive women with abnormal cytology remained 35.6% (13.9‐62.3) in round 1 and 34.2% (17.0‐57.0) in Rounds 2 to 7.

## 4 DISCUSSION

Our analysis suggests that vaccination will lead to a strong decline in the lifetime CIN3+ risk. The estimated decline reached nearly 90% after nonavalent vaccination, translating into an absolute CIN3+ risk reduction from 4.1% to only 0.5%. We expect the absolute lifetime cancer risk to be even much smaller as only a minority of CIN3 cases progress to cancer^40^ and progression rates from CIN3 to cancer are lowest for non‐vaccine types.^41^ To put a lifetime cancer risk below 0.5% into perspective, note that the risk of other gynecological cancers is currently 2.0% for uterus cancer and 1.4% for ovarian cancer,^42^ in which case cervical cancer risk will be dominated by other gynecological cancer risks also in a setting without cervical cancer screening. Nonetheless, the reduction in CIN3+ risk after vaccination strongly depends on the vaccine protection over time. Our sensitivity analysis on the duration of protection indicated that, when vaccination only conferred protection up to screening Round 1 at age 30, the lifetime CIN3+ risks were 2.1% and 1.6% after bivalent with cross‐protection and nonavalent vaccination, respectively. We expect those risks to give rise to similar recommendations for screening and hence this difference in CIN3+ risk can be considered as limited.

The vaccine efficacies estimated in our study were lower than those reported in vaccine trials. In particular, the PATRICIA trial reported an overall efficacy of the bivalent vaccine against CIN3+ of 93%,^3^ substantially higher than 71% estimated by our model. There are, however, some important differences between the PATRICIA trial and the POBASCAM trial used for our model‐based analyses. The PATRICIA trial is a randomized controlled trial in women aged 15 to 25 with intensive surveillance every 6 months whereas the POBASCAM trial is a randomized controlled screening trial in women aged 30 to 60 invited every 5 years. This may lead to differences in the distribution of high‐grade lesions in the two studies because HPV infections have a high probability to be intercepted at CIN2 when kept under close watch. Indeed, the PATRICIA trial reported a much higher proportion of CIN2 cases among CIN2+ than the POBASCAM trial (PATRICIA trial: [186/233] 80% CIN2 among CIN2+, POBASCAM: [295/826] 36% CIN2 among CIN2+). This limits the number of break‐through CIN3 cases and we expect that this holds in particular for types that have a relatively low progression rate and that are not directly targeted by the bivalent vaccine. Therefore, we think that the surveillance intensity may affect the estimated vaccine effect and our conjecture is supported by the observation that in the PATRICIA trial the effect against CIN2+ was only 65%, similar to our estimated effect against CIN2+ of 64%.

Despite the low CIN3+ risk after vaccination, our analysis indicated that screening by HPV in combination with cytology testing for HPV‐positive women may remain feasible. In women vaccinated with a nonavalent vaccine, the CIN3+ risk in HPV‐positive women with abnormal cytology dropped by about 30% to 50% as compared to unvaccinated women, but remained above 10% in both women aged 30 and older women. As 90% of CIN3+ were detected shortly after abnormal cytology,^20^ it is likely that a 10% CIN3+ risk threshold for colposcopy referral can be maintained.^43^ The limited impact of vaccination on the CIN3+ risk in HPV‐positive women after abnormal cytology as compared to the impact of vaccination on the CIN3+ risk itself is related to the specificity of the HPV test. When the background HPV prevalence drops, the specificity of the HPV test increases which weakens the impact of vaccination on the positive predictive value of adjunct cytology. There is still a decrease in positive predictive value because the most aggressive HPV types have been prevented by vaccination, but the estimated decrease was only moderate in our study. If the specificity of the test does not increase when the prevalence of disease decreases, which may be the case for cytology, then the impact on the positive predictive value will be more substantial^44^ which supports a switch from cytology‐based screening to HPV‐based screening in vaccinated cohorts.^45^ Feasibility, however, does not ensure that a program with the same number of screens remains cost‐effective. In vaccinated women, we expect an extension of the interval beyond 5 years to yield a more favorable cost‐effectiveness profile. The low risks presented in our analyses support further initiatives to de‐intensify a screening program in order to optimally balance benefits and harms in women who have been vaccinated at preadolescent age. Such a risk‐based screening strategy would require linkage of vaccination and screening registries and may give rise to some equity concerns. If the screening coverage is high, then a uniform approach may be adopted for both vaccinated and unvaccinated women provided strong herd effects have been demonstrated in unvaccinated women. Evidence on herd effects is currently being collected in several programs.^10^

The present work distinguishes itself from other modeling studies in which lifetime risks are presented^12^, ^14^, ^16^ in that our estimates are derived from a statistical model, driven by HPV‐genotyping and CIN3+ data from a large population‐based screening trial with observations from two consecutive HPV‐based screening rounds with an interval of 5 years. A particular strength is that we applied a newly developed statistical method for dealing with multiple‐type infections.^29^ A main, widely accepted natural history assumption underlying this method is that HPV genotype‐specific infections have a progression risk independent from other HPV genotypes.^30^ Other common methods, i.e. hierarchical and proportional method, lack a formal natural history basis which may bias the estimates. For instance, the hierarchical method overestimates the effect of vaccination as multiple‐type lesions are hierarchically attributed to the most oncogenic types but these are also the HPV genotypes targeted by the vaccines.

Our study has the following limitations. First, we assumed that there are no cohort effects. This may bias estimates of the absolute CIN3+ risk but the effect on the relative decline in risk is likely to be small^46^ as we found little association between type distribution and age.^47^ The absolute CIN3+ risk may be somewhat underestimated because recently the cervical cancer incidence has increased in the Netherlands, in particular in young women.^48^ Second, our statistical model assumes that the HPV incidence does not depend on previous HPV infections (Assumption I). This may have led to an overestimation of the CIN3+ risk as cervical disease is likely to be clustered because of risk factors such as number of lifetime partners, smoking, and so on. Third, the POBASCAM study, which provided the estimates for the present analysis, has some loss to follow‐up among study participants. We believe that loss to follow‐up has only a small effect on the risk estimates as we collected 9 years of follow‐up for every HPV‐positive woman through the national pathology registry (PALGA) and compliance to repeat testing at both 6/18 months and 5 years was 80% to 90%.^20^ Fourth, technical masking of type‐specific infections in the presence of coinfections has not been accounted for, which may occur when a type is present in low copy numbers in a coinfected sample.^49^ Technical masking will lead to an increase in the detected number of type‐specific infections of previously masked genotypes in vaccinated cohorts.^50^ Fifth, we calculated the direct vaccine effect on long‐term CIN3+ risk in vaccinated women and did not consider indirect protective effects in vaccinated and unvaccinated women. We think that for the first vaccinated cohorts, the indirect herd effects in vaccinated women will be limited. Herd effects build up over a longer period of time and substantial herd effects have been observed in the Scottish study,^38^ where the vaccine uptake is at least 85% and the reported efficacies are for those vaccinated at age 12 to 13, 7 years after the start of HPV vaccination. The CIN3+ risk of women after bivalent vaccination was estimated to be only 0.9% when using the vaccine effects observed in the Scottish study, which is 25% lower than our base‐case estimate. Last, we calculated CIN3+ risks under five‐yearly primary HPV screening with adjunct cytology which has been implemented in several countries including Italy, the Netherlands, and Australia. Under co‐testing, CIN3+ rates may be somewhat higher. We calculated that if the HPV test failed to detect 10% of CIN3+, the lifetime CIN3+ risk estimate would lie between 0.5% and 0.8%, suggesting that risk estimates are not strongly influenced by the screening strategy.

To summarize, our study suggests that a considerable drop in the lifetime risk of CIN3+ is to be expected in HPV‐vaccinated women, with a risk reduction up to 90% after nonavalent vaccination with life‐long protection. Primary HPV testing combined with adjunct cytology at intervals of 5 years still seems feasible, although unlikely to be cost‐effective in women that received the nonavalent vaccine. Our calculations support a future de‐intensification of the screening program for vaccinated women provided long‐term vaccine protection.

## CONFLICT OF INTEREST

F. I., B. I. L.‐W., N. J. V., J. A .B., G. R. and J. B. declare no potential conflicts of interest. C. J. L. M. M. is shareholder and part‐time CEO of Self‐Screen BV, a spin‐off company of Amsterdam UMC, location VUMC, which develops, manufactures and licenses a high‐risk HPV assay and methylation marker assays for cervical cancer screening and holds patents on these tests. C. J. L. M. M. has a very small number of shares of MDXHealth and had shares of Qiagen. He has received speaker fees from GlaxoSmithKline, Qiagen and Sanofi Pasteur MSD/Merck and served occasionally on the scientific advisory boards (expert meetings) of these companies. Where authors are identified as personnel of the International Agency for Research on Cancer/World Health Organization, the authors alone are responsible for the views expressed in this article and they do not necessarily represent the decisions, policy or views of the International Agency for Research on Cancer/World Health Organization.

## ETHICS STATEMENT

The POBASCAM trial (Trial registration ID: NTR218) was approved by the Medical Ethics Committee of the VU University Medical Centre (Amsterdam, The Netherlands; no. 96/103) and the Ministry of Public Health (The Hague, The Netherlands; VWS no. 328650). All participants provided written informed consent.

## Supporting information


**Figure S1** Effect of vaccination on the probability of HPV infection.
**Figure S2**: HPV type‐specificrisksofCIN3+ and CIN2 + among HPV type‐positive women.
**Table S1**: Effect of vaccination on the lifetime risksof CIN3+ and CIN2+. The lifetime risksof CIN3+ and CIN2+ are shown with corresponding relativedeclines as compared to no vaccination.
**Table S2**: Effect of vaccination on the CIN3+ and CIN2+ risks in HPV‐positive women. The CIN3+ and CIN2+ risks are shown with corresponding relative declines as compared to no vaccination.
**Table S3**: Effect of vaccination on the CIN3+ and CIN2+ risks in HPV‐positive women with abnormaladjunct cytology. The CIN3+ and CIN2+ risks are shown with corresponding relative declines as compared to no vaccination.

## Data Availability

The data that support the findings of this study are available from the corresponding author upon reasonable request.
