# Supplementary material for: Estimating the direct effect of human papillomavirus vaccination on the lifetime risk of screen‐detected cervical precancer
Source: Int J Cancer. 2020 Jul 28;148(2):320–8. doi: 10.1002/ijc.33207 (PMC7754437; doi:10.1002/ijc.33207)
Supplement: Supplementary file 1 — Figure S1 Effect of vaccination on the probability of HPV infection. Figure S2: HPV type‐specificrisksofCIN3+ and CIN2 + among HPV type‐positive women. Table S1: Effect of vaccination on the lifetime risksof CIN3+ and CIN2+. The lifetime risksof CIN3+ and CIN2+ are shown with corresponding relativedeclines as compared to no vaccination. Table S2: Effect of vaccination on the CIN3+ and CIN2+ risks in HPV‐positive women. The CIN3+ and CIN2+ risks are shown with corresponding relative declines as compared to no vaccination. Table S3: Effect of vaccination on the CIN3+ and CIN2+ risks in HPV‐positive women with abnormaladjunct cytology. The CIN3+ and CIN2+ risks are shown with corresponding relative declines as compared to no vaccination. [file IJC-148-320-s001.pdf]

Supplementary Appendix

Supplement to:

Estimating the direct effect of human papillomavirus vaccination on the lifetime risk of screen-detected cervical precancer

Federica Inturrisi, Birgit I. Lissenberg-Witte, Nienke J. Veldhuijzen, Johannes A. Bogaards, Guglielmo Ronco, Chris J.L.M. Meijer, Johannes Berkhof

Table of Contents

Supplementary Materials & Methods 2

Mathematical formulas for estimating risks 2

Supplementary Tables 4

Table S1: Effect of vaccination on the lifetime risks of CIN3+ and CIN2+, with corresponding relative declines as compared to no vaccination. 4

Table S2: Effect of vaccination on the CIN3+ and CIN2+ risks in HPV-positive women, with corresponding relative declines as compared to no vaccination 4

Table S3: Effect of vaccination on the CIN3+ and CIN2+ risks in HPV-positive women with abnormal adjunct cytology, with corresponding relative declines as compared to no vaccination 5

Supplementary Figures 6

Figure S1: Effect of vaccination on the probability of HPV infection. 6

Figure S2: HPV type-specific risks of CIN3+ and CIN2+ among HPV type-positive women 7

Supplementary Materials & Methods

Mathematical formulas for estimating risks

Let G_i be the set of all women with an HPV infection detected in screening round *i* (*i*=1,…,7). Note that HPV infections in round 2,…,7 are incident infections preceded by a negative HPV test result in the previous round. For the m-th woman in the data, let H_m be the set of all HPV types detected in the cervical smear. H_m is equal to the empty set ∅ if the m-th woman is negative for all high-risk HPV types. The event that any type is detected in the m-th woman can be denoted by $\mathbf{1}_{H_m \neq \emptyset}$ using $\mathbf{1}_Z$ as indicator function for any event Z. For the k-th HPV type ($k = 1, \dots, 14$) within the set [HPV16, HPV18, HPV31, HPV33, HPV35, HPV39, HPV45, HPV51, HPV52, HPV56, HPV58, HPV59, HPV66, HPV68], π_k is the HPV type-specific risk of developing CIN3+ within 9 years.

By Assumptions III and IV in the Methods section, the risk of CIN3+ in HPV-positive women in Round 1 is

$$C_1 = \frac{\sum_{m \in G_1} \mathbf{1}_{H_m \neq \emptyset} \cdot \left\{1 - \prod_{k \in H_m} (1 - \pi_k)\right\}}{\sum_{m \in G_1} \mathbf{1}_{H_m \neq \emptyset}}.$$

Among women with an HPV infection detected in Rounds 2 to 7, the CIN3+ risk is equal to

$$C_2 = \frac{\sum_{m \in \{G_2, \dots, G_7\}} \mathbf{1}_{H_m \neq \emptyset} \cdot \left\{1 - \prod_{k \in H_m} (1 - \pi_k)\right\}}{\sum_{m \in \{G_2, \dots, G_7\}} \mathbf{1}_{H_m \neq \emptyset}}.$$

Risks D_1 and Q_1 are defined in a similar way as C_1, and risks D_2 and Q_2 are defined in a similar way as C_2. More specifically, for D_1 and D_2 the type-specific CIN3+ risk π_k in C_1 and C_2 is replaced by the type-specific risk of both CIN3+ and abnormal cytology in women positive for the k-th HPV-type, and for Q_1 and Q_2 the type-specific risk π_k is replaced by the type-specific risk of abnormal cytology in women positive for the k-th HPV-type.

Denote by VE_k the vaccine efficacy of the k-th HPV genotype. Then, after vaccination, the probability of a positive HPV result in screening round i $(i = 1, \ldots, 7)$ becomes

$$P_i = \frac{\sum_{m \in G_i} \mathbf{1}_{H_m \neq \emptyset} \cdot \{1 - \prod_{k \in H_m} VE_k\}}{\sum_{m \in G_i} 1}.$$

After vaccination, the risk of developing CIN3+ after a positive result in Round 1 becomes

$$C_1 = \frac{\sum_{m \in G_1} \mathbf{1}_{H_m \neq \emptyset} \cdot \{1 - \prod_{k \in H_m} (1 - (1 - VE_k) \cdot \pi_k)\}}{\sum_{m \in G_1} \mathbf{1}_{H_m \neq \emptyset} \{1 - \prod_{k \in H_m} VE_k\}},$$

and the CIN3+ risk after a positive result in Rounds 2 to 7 becomes

$$C_2 = \frac{\sum_{m \in \{G_2, \ldots, G_7\}} \mathbf{1}_{H_m \neq \emptyset} \{1 - \prod_{k \in H_m} (1 - (1 - VE_k) \cdot \pi_k)\}}{\sum_{m \in \{G_2, \ldots, G_7\}} \mathbf{1}_{H_m \neq \emptyset} \{1 - \prod_{k \in H_m} VE_k\}}.$$

D_1, D_2, Q_1 and Q_2 were re-estimated in a similar way as shown for $C_1$and C_2.

Supplementary Tables

Table S1: Effect of vaccination on the lifetime risks of CIN3+ and CIN2+, with corresponding relative declines as compared to no vaccination.

	Scenario	CIN3+		CIN2+	
		Risk (95% CI)	Relative decline (95% CI)	Risk (95% CI)	Relative decline (95% CI)
Lifetime	no vaccination	4.1 (3.5 to 4.9)	ref	6.5 (5.6 to 7.3)	ref
	2/4vHPV	1.9 (1.4 to 2.4)	53.5 (43.7 to 62.2)	3.5 (2.8 to 4.1)	46.8 (38.9 to 54.8)
	2vHPV + cross-protection	1.2 (0.9 to 1.5)	70.5 (64.4 to 78.0)	2.3 (1.8 to 2.8)	64.3 (57.7 to 70.4)
	9vHPV	0.5 (0.2 to 0.7)	88.5 (82.4 to 94.3)	1.1 (0.8 to 1.6)	82.7 (76.3 to 88.0)
Residual lifetime after an HPV-negative result in Round 1	no vaccination	1.4 (1.0 to 1.9)	ref	2.8 (2.2 to 3.4)	ref
	2/4vHPV	0.8 (0.5 to 1.2)	42.5 (25.2 to 55.2)	1.9 (1.4 to 2.4)	32.9 (22.2 to 44.3)
	2vHPV + cross-protection	0.5 (0.3 to 0.7)	64.7 (51.7 to 75.1)	1.3 (0.9 to 1.7)	54.7 (45.2 to 64.8)
	9vHPV	0.2 (0.1 to 0.4)	85.1 (71.4 to 94.8)	0.7 (0.4 to 1.0)	76.2 (65.4 to 86.0)
Lifetime with vaccine protection up to Round 1 at age 30	no vaccination	4.1 (3.5 to 4.9)	ref	6.5 (5.6 to 7.3)	ref
	2/4vHPV	2.5 (1.9 to 3.1)	40.1 (31.4 to 49.2)	4.3 (3.6 to 5.1)	33.6 (26.6 to 40.4)
	2vHPV + cross-protection	2.1 (1.6 to 2.6)	49.7 (41.5 to 58.5)	3.8 (3.1 to 4.4)	42.0 (35.1 to 48.8)
	9vHPV	1.6 (1.2 to 2.1)	60.6 (51.7 to 69.0)	3.2 (2.6 to 3.8)	50.9 (43.5 to 58.1)

Table S2: Effect of vaccination on the CIN3+ and CIN2+ risks in HPV-positive women, with corresponding relative declines as compared to no vaccination.

	Scenario	CIN3+		CIN2+	
		Risk (95% CI)	Relative decline (95% CI)	Risk (95% CI)	Relative decline (95% CI)
Round 1	no vaccination	25.0 (20.3 to 29.1)	ref	34.6 (29.9 to 39.7)	ref
	2/4vHPV	15.0 (9.5 to 19.8)	39.9 (25.6 to 57.8)	21.8 (16.2 to 28.0)	36.9 (22.7 to 49.8)
	2vHPV + cross-protection	12.5 (7.4 to 16.4)	50.0 (36.8 to 68.1)	18.5 (13.2 to 24.9)	46.4 (30.6 to 60.2)
	9vHPV	6.8 (1.8 to 11.0)	72.9 (57.6 to 92.5)	11.9 (6.1 to 19.3)	65.5 (45.7 to 81.8)
Rounds 2 to 7	no vaccination	9.0 (6.6 to 11.9)	ref	17.9 (14.6 to 21.3)	ref
	2/4vHPV	6.8 (4.5 to 9.8)	24.9 (3.5 to 41.9)	15.7 (12.0 to 19.5)	12.3 (−0.7 to 26.3)
	2vHPV + cross-protection	5.1 (3.2 to 7.7)	42.8 (21.2 to 59.5)	13.1 (9.5 to 16.7)	26.6 (12.2 to 42.7)
	9vHPV	3.1 (1.1 to 6.1)	65.5 (34.9 to 87.9)	9.9 (5.8 to 14.6)	44.9 (20.4 to 66.6)

Table S3: Effect of vaccination on the CIN3+ and CIN2+ risks in HPV-positive women with abnormal adjunct cytology, with corresponding relative declines as compared to no vaccination.

	Scenario	CIN3+		CIN2+	
		Risk (95% CI)	Relative decline (95% CI)	Risk (95% CI)	Relative decline (95% CI)
Round 1	no vaccination	52.0 (40.6 to 61.1)	ref	65.9 (54.5 to 73.2)	ref
	2/4vHPV	41.4 (26.3 to 53.6)	20.3 (3.4 to 42.2)	54.8 (41.4 to 69.1)	16.8 (−1.4 to 31.5)
	2vHPV + cross-protection	40.1 (23.7 to 54.3)	22.9 (1.9 to 47.2)	53.8 (37.7 to 71.2)	18.5 (−3.6 to 36.6)
	9vHPV	28.2 (6.6 to 50.8)	45.8 (5.4 to 86.3)	35.6 (13.9 to 62.3)	46.0 (6.1 to 77.2)
Rounds 2 to 7	no vaccination	20.3 (14.1 to 30.0)	ref	40.2 (32.0 to 50.9)	ref
	2/4vHPV	18.0 (10.0 to 29.3)	11.5 (−18.7 to 40.4)	40.3 (29.4 to 52.9)	−0.2 (−20.4 to 20.9)
	2vHPV + cross-protection	15.4 (8.0 to 25.9)	24.3 (−7.9 to 53.8)	38.4 (26.0 to 52.8)	4.3 (−22.1 to 29.5)
	9vHPV	13.9 (3.2 to 27.8)	31.8 (−25.4 to 82.9)	34.2 (17.0 to 57.0)	14.8 (−34.3 to 55.1)

Supplementary Figures

Figure S1: Effect of vaccination on the probability of HPV infection.

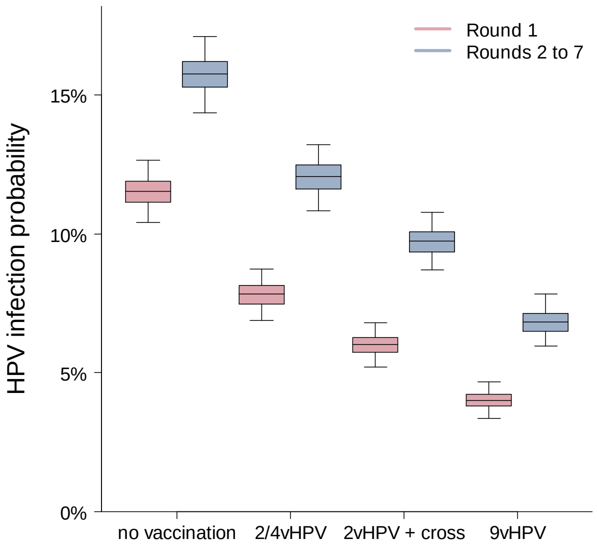

The probabilities of HPV infection are estimates needed for the estimation of the lifetime risks of CIN3+ and CIN2+. The probability of a prevalent HPV infection in Round 1 is shown in blue and the probability of an incident HPV infection in Rounds 2 to 7 is shown in red. Separate estimates are presented for the no vaccination scenario and for the three scenarios bivalent/quadrivalent (2/4vHPV), bivalent with cross-protection (2vHPV + cross), and nonavalent (9vHPV) vaccination.

Figure S2: HPV type-specific risks of CIN3+ and CIN2+ among HPV type-positive women.

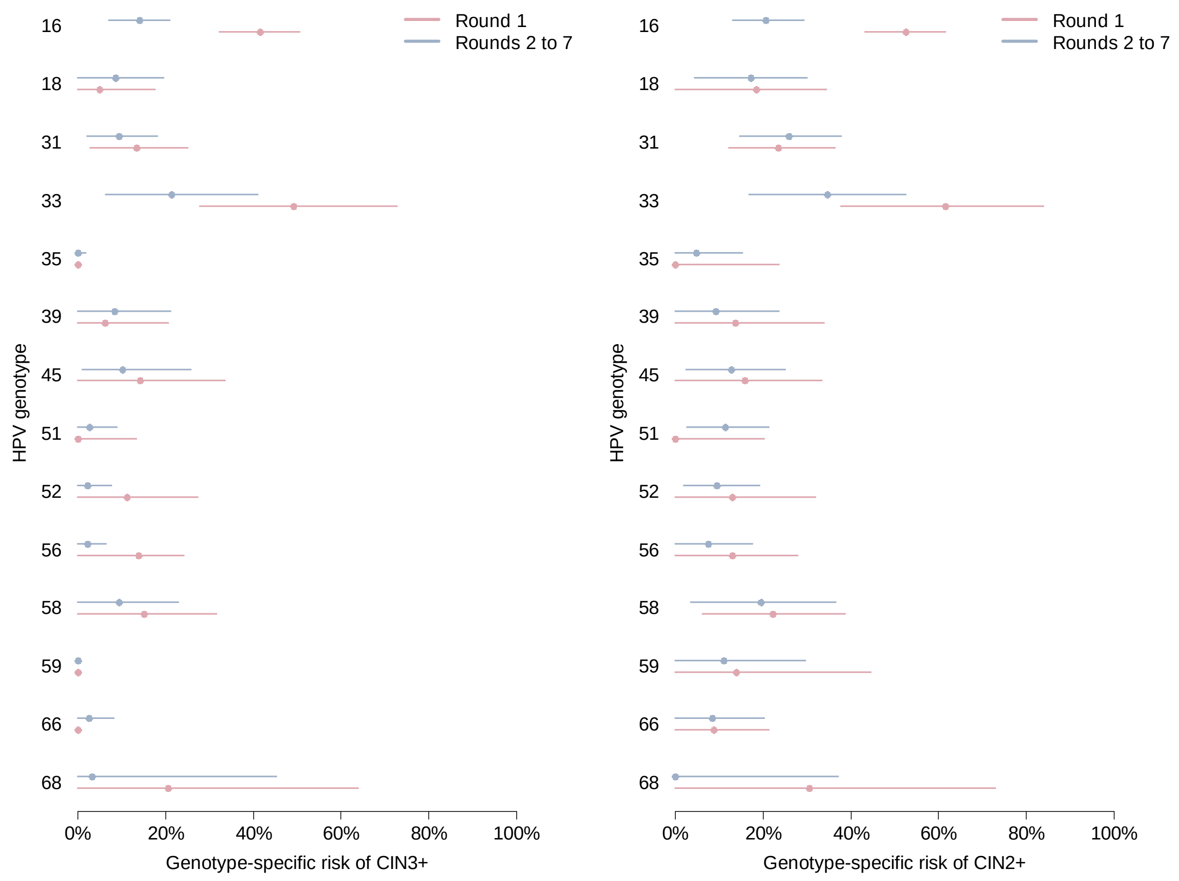

The HPV type-specific risks of CIN3+ (left) and CIN2+ (right) among HPV type-positive women are estimates needed for the estimation of the lifetime risks of CIN3+ and CIN2+. The HPV type-specific risks in Round 1 and in Rounds 2 to 7 are shown in blue and red respectively, for 14 high-risk HPV genotypes (HPV 16, 18, 31, 33, 35, 39, 45, 51, 52, 56, 58, 59, 66, and 68).
